# ECMO Support in Pediatric Populations with the Newborn ECMOLife Centrifugal Pump

**DOI:** 10.3390/medicina61030493

**Published:** 2025-03-13

**Authors:** Carlo Pace Napoleone, Ignazio Condello, Maria Teresa Cascarano, Enrico Aidala, Licia Peruzzi, Isabella Molinari, Cristina Rivoldini, Maria Stella Di Carlo, Stefania Iannandrea, Enrico Bonaveglio

**Affiliations:** 1 Department of Pediatric and Congenital Cardiac Surgery, Regina Margherita Children’s Hospital, 10024 Torino, Italy; 2 Department of Cardiac Surgery, Anthea Hospital, GVM Care & Research, Via Camillo Rosalba 35/37, 70124 Bari, Italy

**Keywords:** extracorporeal membrane oxygenation, mechanical circulatory support, pediatric cardiac failure, pediatric respiratory failure, magnetic levitation

## Abstract

*Background and Objectives*: Pediatric ECMO is a valid support mechanism for refractory cardiac and/or respiratory failure. Magnetic levitation technology applied to the centrifugal pump has reduced the hemolysis caused by this procedure, which can be particularly dangerous, especially in neonates and small children. ECMOLife, a new magnetic levitation centrifugal pump, has been introduced for these patients. *Materials and Methods*: Four patients were supported with the ECMOLife System in a newborn setting, with veno-venous application in two cases and veno-arterial in the other two. All parameters related to pump functioning, anticoagulation, hemolysis, and inflammation were recorded for the duration of the support. *Results*: All patients survived the procedure, in three cases achieving recovery, while one veno-arterial ECMO was switched to VAD, and then the patient underwent heart transplantation. All recorded parameters were compatible with clinical conditions. In particular, free haemoglobin was close to 0 g/L in all recorded samples. The possibility of monitoring pump functioning parameters, venous and arterial O2 saturation, and venous and arterial pressures creates an opportunity to check the adequacy of mechanical support for the clinical condition of the patient. *Conclusions*: This is the first reported experiment in a newborn setting with ECMOLife mechanical support. At present, ECMOLife represents the only system with a newborn and pediatric pump, allowing for the continuous monitoring of perfusion and hemodynamic parameters, with a large number of facilities for transportation available.

## 1. Introduction

Extracorporeal membrane oxygenation (ECMO) is a supportive strategy used to manage patients with refractory cardiac and/or respiratory failure despite maximal medical therapy [1]. It was first introduced in 1972 for the treatment of a critically ill adult patient with respiratory failure, but since then, the application of ECMO has expanded to treat patients with either cardiac failure, respiratory failure, or both. ECMO usage has progressively expanded to include pediatric patients, with the first neonatal survivor in 1976. This solved problems peculiar to these patients, like those involving vessel dimensions, a necessity for adequate flow in low-body-weight cases, and fragile blood components [2]. ECMO-related complications, attributed with greater prevalence to centrifugal pumps, include hypertension, bleeding, and hyperbilirubinemia, which are in turn accelerated by hemolysis and which have been associated with increased mortality. The latest generation of centrifugal pumps, using magnetic levitation technology, exhibit mechanical properties which may have overcome the limitations of first-generation devices, reducing shear stress and localized heat generation and having a beneficial effect on hemolysis [3,4]. This can allow for a reduction in collateral effects related to ECMO procedures, which can be beneficial especially in neonates and small children, like reductions in acute kidney and multi-organ injury rates, with a positive impact on the procedure outcome [5]. The PediVas (Abbott, Chicago, IL, USA) was, for a long time, the first and only neonatal/pediatric magnetically suspended centrifugal pump with a static prime of 14 mL and with one-quarter-inch pump connectors [6]. A new product, the ECMOLife system (Eurosets, Medolla, Italy), has been proposed as an alternative to PediVas based on real magnetic levitation technology and is available in both adult and neonatal/pediatric configurations [7]. It is a magnetically levitated centrifugal pump comprising a rotating impeller with six blades encased in a polycarbonate housing. The pump can provide blood flow rates ranging from 0 to 3 L/min with a maximum of 4500 RPM. The ECMOLife console includes probes for measuring the delivered ECMO flow, pre-oxygenator oxygen saturation, and haemoglobin concentration and for detecting gaseous microemboli.

The primary objective of this study is to evaluate the efficacy and safety of the ECMOLife system, a new magnetically levitated centrifugal pump, in a pediatric population. This system represents a significant advancement in ECMO technology in that it may potentially reduce the complications associated with traditional centrifugal pumps, such as hemolysis, which can be particularly detrimental in neonates and small children. We aim to conduct the following investigations:

1. Assessment of the clinical outcomes of pediatric patients undergoing ECMO with the ECMOLife system.
2. Evaluation of the system’s ability to minimize common ECMO-related complications, such as hemolysis and mechanical failure.
3. Exploration of the feasibility and safety of using this system in both hospital and transport settings, given its design enhancements for mobility and stability.

By achieving these objectives, we hope to contribute valuable data on the performance of new ECMO technology and its potential to improve outcomes for critically ill pediatric patients. We present the clinical experiences of four pediatric patients treated with the neonatal ECMOLife system, which are, to the best of our knowledge, the first to be reported in the literature.

## 2. Materials and Methods

Four pediatric patients, whose parents gave written consent for the use of their clinical data for publication, were evaluated. This study was approved by the Ethical Institutional Board of Regina Margherita Children’s Hospital of Torino, Italy (approval number 0001044, on 16 December 2024).

Patients considered for this study were selected based on the following inclusion criteria:

1. Age and Weight: Neonates and pediatric patients ranging from birth to 12 years of age, with body weights appropriate for the ECMOLife system’s operational parameters (from 2 to 40 kg).
2. Clinical Need for ECMO: Patients included must have exhibited refractory cardiac and/or respiratory failure not responding to maximum conventional medical management, indicating a clear need for extracorporeal life support as determined by the attending pediatric cardiologist or intensivist.
3. Previous Interventions: All patients must have undergone standard medical interventions appropriate for their condition (e.g., mechanical ventilation, inotropic support) without sufficient clinical improvement before ECMO consideration.
4. No Contraindications for ECMO: Absence of any recognized contraindications to ECMO treatment, such as irreversible brain damage, terminal illnesses without the option for recovery, or severe coagulopathies not correctable with medical management.
5. Consent: Written informed consent had to be obtained from the parents or legal guardians of all patients.

These criteria ensured that the study involved patients who were most likely to benefit from the ECMOLife System while maintaining strict ethical standards for patient selection and care. The patients underwent ECMO implantation with the ECMOLife system in a newborn setting at Regina Margherita Children’s Hospital, Torino, Italy, from October to December 2023. This setting comprised the neonatal centrifugal pump, the neonatal oxygenator ECMO A.L.ONE (Eurosets, Medolla, Italy) and a PVC circuit ¼-¼. The whole system was phosphorilcoline-coated, DEHP-free, and Latex-free. We started the anticoagulation protocol with heparin IV infusion only 12 h after ECMO initiation. Anticoagulation management involves regular monitoring of activated clotting time (ACT) and anti-Xa levels to ensure appropriate anticoagulation while minimizing the risk of bleeding. For ACT, the typical target range in pediatric patients is 180–220 s. This range may be adjusted based on individual patient factors, such as age, bleeding risk, and thrombosis risk. For neonates or those with a higher risk of bleeding, the lower end of this range may be preferred, while slightly higher values may be used in patients with an increased risk of clot formation. For anti-Xa levels, the usual target range is 0.3–0.7 IU/mL. Anti-Xa levels provide a more precise measure of heparin’s anticoagulant effect, with the lower end of the range (0.3 IU/mL) being sufficient for most pediatric patients without increasing bleeding risk. The upper end (0.7 IU/mL) may be necessary for patients at higher risk of thrombosis.

### System Specifications

**Figure 1:**
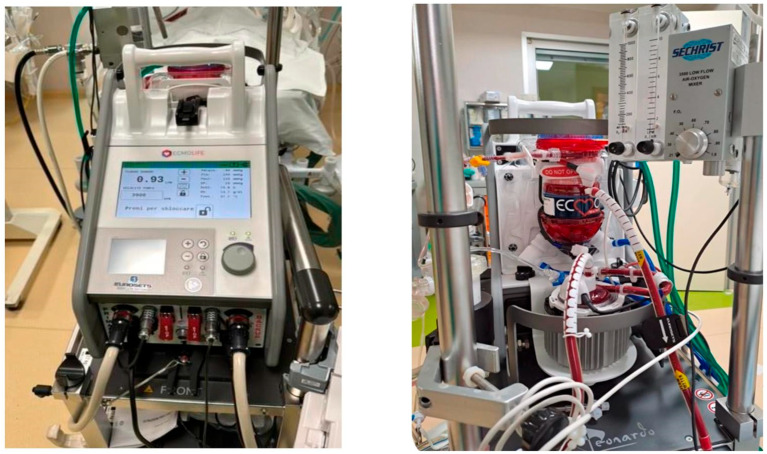
Perioperative use of ECMOLIFE System.

The ECMOLife System used in this study is designed for neonatal and pediatric patients, and it provides the flexibility to support a wide range of patient sizes. However, specific details regarding the system’s operational parameters are essential for optimizing its use in different clinical settings. The recommended patient weight range for this configuration typically spans from neonates to small pediatric patients, with an upper limit based on the ability to provide adequate flow rates (Figure 1).

- Blood Flow Range: The ECMOLife pump is capable of delivering blood flows from 0 to 3 L/min, which is suitable for neonatal and small pediatric patients. With an oxygenator, the Newborn ECMOLife preconnected to the suggested pump allows blood to flow from 0 to 1.5 L/min.
- RPM: The maximum rotational speed of the pump reaches 4500 RPM, allowing precise control of flow based on patient size and needs.
- Cart Size: The ECMOLife system is compact and mobile, making it convenient for use in both hospital and transport settings. Its compact cart size allows for easier mobility within intensive care units, as well as for external transport in ambulances and helicopters.
- Automated Parameters: The system includes several automated monitoring features, such as real-time measurement of ECMO flow, negative pressure on the drainage line, pre- and post-oxygenator pressure with relative pressure drop, pre-oxygenator oxygen saturation, haemoglobin concentration, and detection of gaseous microemboli. These features enable continuous surveillance of both patient and circuit parameters, reducing the manual workload on the clinical team.

## 3. Case Reports

### 3.1. Summary of Pediatric ECMO Patients Supported by the ECMOLife System (Table 1)

### 3.2. Case 1

The first patient was a 3.5 kg neonate with severe respiratory failure due to a left diaphragmatic hernia, who underwent veno-venous ECMO support through the right jugular vein using a 13Fr Avalon Elite Bi-Caval Dual-Lumen Catheter (Maquet Cardiopulmonary, Rastatt, Germany) a few hours after delivery. The diaphragm was successfully repaired on the second day of life, and the patient was supported with ECMO for 6 days before weaning (Table 2a,b).

### 3.3. Case 2

The second case was a 4-month-old baby weighing 6.7 kg who underwent anatomical correction of the double-outlet right ventricle. On postoperative day 3, the patient experienced severe viral pneumonia due to meta-pneumovirus and rhino-entherovirus with a superinfection from pseudomonas aeruginosas with consequent refractory respiratory failure. A veno-venous ECMO was then positioned through the right jugular vein, using a 16 fr. Avalon Elite Bi-Caval Dual-Lumen Catheter and Newborn ECMOLife. Respiratory recovery was obtained after aggressive antibiotic therapy and protective ventilation, and the patient was successfully weaned from ECMO on the 12th post-implantation day and discharged after an uneventful course of treatment (Table 3a,b).

### 3.4. Case 3

The third patient was affected by a hypoplastic left heart complex with severe left ventricular hypoplasia, a ventricular septal defect, left ventricular myocardial non-compaction, and aberrant retro oesophagal right subclavian artery. The baby underwent neonatal pulmonary artery banding at 15 days of age. At the age of 5 months (4.7 kg), he underwent comprehensive two-stage single-ventricle surgical palliation using Damus–Kay–Stansel aorto-pulmonary connection, Glenn procedure, tricuspid valve repair, and right aberrant subclavian artery reimplantation. On postoperative day 1, a central veno-arterial ECMO was initiated because of severe systemic right ventricular failure. A Newborn ECMOLife and a Newborn A.L.ONE ECMO oxygenator were used. The patient was listed for status 1 heart transplantation and underwent successful ABO-incompatible heart transplantation on the ninth day after ECMO implantation. The remaining postoperative course of treatment was uneventful (Table 4a,b).

### 3.5. Case 4

The fourth patient was a 4 year-old (18 kg) with a diagnosis of acute viral myocarditis who underwent rescue advanced cardiac life support (ECLS) using Newborn ECMOLife with A.L.ONE ECMO oxygenator and ¼-¼ circuit through femoral–femoral cannulation as a bridge to decision. Peripheral cannulation was obtained with a 15 Fr venous cannula and a 14 Fr arterial cannula (Biomedical, Medtronic, Minneapolis, USA). During the second post-ECMO day, for evidence of left ventricular volume overload, it was necessary to add a 12 Fr left ventricular vent through median sternotomy, with effective reduction of pulmonary impairment and hemodynamic stabilization. The necessity of using the Newborn ECMO setting in patient weighing 18 kg was due to the unavailability of a larger one because all other ECMO machines were running on other patients. This prompted us to use the only available machine in a newborn setting, although it was evidently undersized for the patient. Under these conditions, at the beginning of support, it was possible to obtain a maximum flow of 1200 mL/min, while the estimated optimal flow for the patient was about 2200 mL/min. Nevertheless, the flow obtained at a mean of 3800 rpm/min was acceptable and caused an immediate reduction in lactate and hemodynamic stabilization. Two days later, it was necessary to insert a left atrial vent that increased the flow to a mean of 1500 mL/min. Eight days later, the mechanical support was switched to the left ventricular Excor Berlin Heart VAD as a long-term device for the bridge to transplantation. Finally, the patient underwent heart transplantation after 8 months from the initial ECLS with ECMOLife (Table 5a,b).

## 4. Results

All patients were successfully supported with Newborn ECMOLife, and hemodynamic and respiratory stabilization was promptly obtained soon after implantation. Daily checks of free plasma haemoglobin gave values close to 0 in all cases. It is important to note that in the fourth patient, who received an evidently undersized circuit, values of negative pressure oscillating between −126 and −140 mmHg were recorded on the venous return, while the positive pressure on the arterial line oscillated between 96 and 136 mmHg. In these conditions, the shear stress on red blood cells usually accelerates the hemolysis. The consequent increase in free plasma haemoglobin has a deleterious effect on the splanchnic organs, above all on the kidneys. Incredibly, also in this patient, the free haemoglobin recorded during the 8 days of support oscillated between 0 and 0.05 g/L. In all four patients, no adverse events were recorded after ECMO running.

## 5. Discussion

This is the first pediatric experience reported in the literature with Newborn ECMOLife (Eurosets, Medolla, Italy). Our impression is that the concept of magnetic levitation applied to centrifugal pumps is of crucial importance in reducing blood shear stress and hemolysis. The outcome of ECMO in the pediatric population in terms of organ function and survival strongly depends on the integrity of blood components [4]. The most impressive consideration in all four cases presented in this paper is that the level of free haemoglobin concentration remained close to 0 during the entire period of ECMO support. Furthermore, the system gives the possibility of checking in real time the in- and out-pressures and parameters like haemoglobin concentration and mixed venous oxygen saturation. It is then possible to have important hemodynamic and pump performance data at every single moment during ECMO support. The ECMOLife is also very compact, easy to move inside the hospital, and has many supports that are certified for extra-hospital transport in the ambulance, helicopter and even by drone [8]. Moreover, the availability of an integrated independent backup motor increases the level of security, especially in extra-hospital environments. The magnetic levitation technology of the ECMOLife pump was designed with this in mind, offering a reduction in shear stress on blood components and providing a more gentle circulation compared to traditional pumps. In this study, no chattering or suction events were observed during the ECMO runs. The system maintained stable negative pressures, which are crucial in preventing hemolysis. Excessive negative pressures or fluctuations can lead to red blood cell damage, and the lack of such events in our cases suggests that the ECMOLife system performed reliably under various clinical conditions. The ECMOLife system includes several key features aimed at enhancing both patient safety and ease of use. The system is equipped with pressure-monitoring capabilities, allowing for real-time adjustments to maintain stable pressures in both the venous and arterial circuits. This contributes significantly to reducing the risk of complications like excessive negative pressures, which can lead to hemolysis. The system includes an automatic detection system for gaseous microemboli, which immediately alerts the clinical team and allows for prompt intervention to avoid serious complications. The hardware is designed to be user-friendly, with a straightforward interface that facilitates real-time monitoring of key parameters such as blood flow, pressure, and oxygenation. Additionally, the system includes a backup motor to enhance safety, particularly in the event of power or mechanical failure, and it is equipped with multiple alarms for critical conditions, ensuring patient safety at all times. From our initial experience, the compact design of the system was an advantage, particularly in the intensive care unit and for transport between facilities.

### Limitations of the Study

This study, while pioneering in its use of the ECMOLife System in a pediatric setting, is not without limitations that must be acknowledged. They include the following. (a) Small Sample Size: This study involved a small cohort of only four pediatric patients, which limits the generalizability of the findings. Larger studies are necessary to validate the outcomes observed in this initial trial. (b) Single-Center Design: As the study was conducted at a single institution, its results might not be reflective of broader clinical practice. Multi-centre studies would be beneficial to assess the system’s performance across different clinical settings and patient populations. (c) Lack of a Control Group: The absence of a control group using a different ECMO system or traditional management approaches limits the ability to directly compare the effectiveness of the ECMOLife System against existing technologies. (d) Patient Heterogeneity: The patients included in the study had varied underlying conditions and ECMO requirements, which could have affected the outcomes and the applicability of the findings to other populations. (e) Technical Challenges: This study encountered specific challenges related to the size and flow requirements for certain patients, particularly in adapting the newborn settings for larger pediatric patients, which might have impacted the efficacy and safety outcomes [9]. (f) Follow-up Duration: This study’s short-term follow-up period did not allow for the assessment of long-term outcomes and potential late complications associated with the use of the ECMOLife system in pediatric patients. Despite this exciting first experience, further studies including a larger number of patients will be necessary to confirm the superiority of the Newborn ECMOLife over other centrifugal pumps sharing the same concept of magnetic levitation.

## 6. Conclusions

The initial clinical application of the ECMOLife system in pediatric patients shows promising safety and efficacy outcomes. In this study, all enrolled patients experienced stable and effective mechanical support without immediate adverse events, suggesting that the ECMOLife system can provide reliable extracorporeal support for severe cardiac and respiratory failure in pediatric settings. Notably, the system’s magnetic levitation technology appears to significantly reduce complications typically associated with traditional ECMO, such as hemolysis, with free haemoglobin levels remaining consistently low across all cases. This indicates minimal blood damage, which may reduce the risk of organ dysfunction. The ECMOLife system also demonstrated versatility and mobility, facilitating its use across various clinical environments from intensive care units to transport scenarios, thereby enhancing adaptability in patient management. Although definitive conclusions about long-term outcomes and broader applicability require further study, the immediate clinical improvements observed suggest that the ECMOLife system could improve the standard of care in pediatric ECMO therapy. Future research involving larger, controlled studies is needed to more comprehensively assess the effectiveness and safety of this system in a broader pediatric population.

## Figures and Tables

**Table 1:** Summary of Pediatric ECMO.

Case	Age/Weight	Diagnosis	ECMO Type	Support Duration	Outcome
1	3.5 kg Neonate	Severe respiratory failure (hernia repair)	Veno-Venous	6 Days	Weaned, recovery achieved
2	4 months/6.7 kg	Viral pneumonia & Pseudomonas infection	Veno-Venous	12 Days	Weaned, recovery achieved
3	5 months/4.7 kg	Hypoplastic left heart, systemic failure	Veno-Arterial	9 Days	Transitioned to heart transplant
4	4 yrs/18 kg	Acute viral myocarditis, LV overload	Veno-Arterial	8 Days	Switched to VAD → Heart transplant

**Table 2:** (**a**) Perioperative technical parameters (Case 1). (**b**) Perioperative laboratory parameters (Case 1).

(a)							
	Blood Flowm L/min	RPM	Press Drain mmHg	Press IN mmHg	Press OUT mmHg	DELTA P mmHg	SVO_2_ %
T 0	580	3150	−100	96	83	13	93
DAY 1	520	3400	−100	115	100	15	95
DAY 2	500	3550	−127	115	95	20	95
DAY 3	500	3850	−133	75	59	16	96
DAY 4	460	3400	−123	94	78	16	85
DAY 5	450	3300	−123	81	67	14	88
DAY 6	420	3100	−105	83	63	20	72
**(b)**							
	**Free Plasma Hb g/L**	**Anti Xa** **UI/mL**	**Lact** **mmol/L**	**CRP** **mg/L**	**PLT** **10^9^/L**	**D-Dimer** **ng/mL**	**Fibrinogen** **mg/dL**
T 0	0.0	0.5	5.7	7.2	80	20,645	165
DAY 1	0.04	0.54	4.6	19.6	65	18,166	156
DAY 2	0.0	0.34	1.6	11.3	87	12,656	159
DAY 3	0.03	0.48	1.7	8.7	68	13,305	145
DAY 4	0.0	0.41	2.2	4.9	61	27,878	101
DAY 5	0.03	0.40	2.2	2.6	34	37,349	68
DAY 6	0.0	0.33	1.5	1.7	222	53,474	100

**Table 3:** (**a**) Perioperative technical parameters (Case 2). (**b**) Perioperative laboratory parameters (Case 2).

(a)							
	Blood Flow mL/min	RPM	Press Drain mmHg	Press IN mmHg	Press OUT mmHg	DELTA P mmHg	SVO_2_ %
T 0	900	3900	−103	100	80	20	77
DAY 1	900	3900	−112	150	130	20	80
DAY 2	900	3900	−87	140	120	20	80
DAY 3	950	4100	−82	175	152	23	72
DAY 4	950	3950	−65	170	145	25	86
DAY 5	1000	3950	−65	155	135	20	82
DAY 6	1000	3950	−65	165	145	20	89
DAY 7	950	3950	−65	155	135	20	89
DAY 8	950	3950	−70	150	128	22	89
DAY 9	950	3950	−60	155	130	25	77
DAY 10	970	3950	−60	155	135	20	75
DAY 11	950	3950	−92	158	138	20	74
**(b)**							
	**Free Plasma Hb g/L**	**Anti Xa** **UI/mL**	**Lact** **mmol/L**	**CRP** **mg/L**	**PLT** **10^9^/L**	**D-Dimer** **ng/mL**	**Fibrinogen** **mg/dL**
T 0	0.0	0.54	1.9	19.6	670	4285	279
DAY 1	0.04	0.43	0.8	10.2	335	6514	272
DAY 2	0.0	0.41	0.8	10.9	256	7414	458
DAY 3	0.03	0.43	1.0	18	168	18,883	375
DAY 4	0.0	0.44	0.9	24	137	22,321	324
DAY 5	0.03	0.51	0.7	31.5	123	42,275	299
DAY 6	0.0	0.40	0.5	29	88	64,430	315
DAY 7	0.0	0.54	1.0	23.5	79	60,392	307
DAY 8	0.0	0.36	1.0	13.5	98	28,873	242
DAY 9	0.0	0.52	1.5	9.7	81	38,363	163
DAY 10	0.03	0.58	1.4	12.3	79	29,103	157
DAY 11	0.04	0.56	1.0	12.9	76	19,454	143

**Table 4:** (**a**) Perioperative technical parameters (Case 3). (**b**) Perioperative laboratory parameters (Case 3).

(a)							
	Blood Flow mL/min	RPM	Press Drain mmHg	Press IN mmHg	Press OUT mmHg	DELTA P mmHg	SVO_2_ %
T 0	620	4000	−36	270	255	15	90
DAY 1	600	4000	−35	269	255	14	87
DAY 2	630	4000	−34	275	260	15	88
DAY 3	520	3600	−30	260	248	12	90
DAY 4	520	3500	−23	204	195	9	87
DAY 5	500	3500	−23	207	193	14	85
DAY 6	560	3800	−25	244	232	12	88
DAY 7	580	3900	−35	233	240	7	91
DAY 8	520	3800	−30	236	232	4	93
DAY 9	600	4000	−33	259	258	1	89
**(b)**							
	**Free Plasma Hb g/L**	**Anti Xa** **UI/mL**	**Lact** **mmol/L**	**CRP** **mg/L**	**PLT** **10^9^/L**	**D-Dimer** **ng/mL**	**Fibrinogen** **mg/dL**
T 0	0.0	0.25	1.3	27.47	70	4659	122
DAY 1	0.0	0.29	1.6	9.3	60	7377	106
DAY 2	0.0	0.34	1.4	7.9	137	8025	115
DAY 3	0.0	0.45	2.2	5.3	63	9632	129
DAY 4	0.0	0.41	2.0	3.2	52	11,516	138
DAY 5	0.0	0.42	1.9	2.2	169	14,214	161
DAY 6	0.0	0.38	1.7	3.1	130	14,347	138
DAY 7	0.0	0.32	1.0	4.6	90	34,066	139
DAY 8	0.0	0.35	0.8	16.8	40	35,908	166
DAY 9	0.0	0.31	0.9	15	69	63,336	179

**Table 5:** (**a**) Perioperative technical parameters (Case 4). (**b**) Perioperative laboratory parameters (Case 4).

(a)							
	Blood Flow mL/min	RPM	Press Drain mmHg	Press IN mmHg	Press OUT mmHg	DELTA P mmHg	SVO_2_ %
T 0	1200	3800	−132	120	96	26	63
DAY 1	1300	3900	−132	145	119	26	55
DAY 2	1550	4000	−130	171	131	39	86
DAY 3	1500	4000	−126	177	136	40	91.3
DAY 4	1550	4000	−142	149	114	45	82.2
DAY 5	1540	4000	−139	159	127	39	85.7
DAY 6	1400	4000	−140	156	117	35	82.4
DAY 7	1370	4000	−140	160	117	43	87.1
DAY 8	1340	4000	−140	158	110	48	87
**(b)**							
	**Free Plasma Hb g/L**	**Anti Xa** **UI/mL**	**Lact** **mmol/L**	**CRP** **mg/L**	**PLT** **10^9^/L**	**D-Dimer** **ng/mL**	**Fibrinogen** **mg/dL**
T 0	0.0	0.62	1	33.9	288	1887	374
DAY 1	0.04	0.44	1	53.1	229	2660	306
DAY 2	0.0	0.59	2.9	84.5	218	2169	367
DAY 3	0.03	0.46	1.4	125.4	104	1414	450
DAY 4	0.0	0.45	0.9	151.7	89	7985	565
DAY 5	0.03	0.50	0.7	132.1	88	21,707	551
DAY 6	0.0	0.46	1.8	91	143	31,602	556
DAY 7	0.0	0.56	1.3	90.2	104	85,164	285
DAY 8	0.0	0.57	0.9	58.2	84	115,990	113

## Data Availability

The data presented in this study are available upon request from the corresponding author due to ethical considerations.
